# Concomitant Subarachnoid Hemorrhage and Ischemic Stroke Associated With Ruptured Aneurysms: Case Reports

**DOI:** 10.7759/cureus.85763

**Published:** 2025-06-11

**Authors:** Patrice Finet, Michel Bojanowski

**Affiliations:** 1 Neurosurgery, UCLouvain, Bruxelles, BEL; 2 Neurosurgery, Centre Hospitalier de l'Université de Montréal (CHUM), Montreal, CAN

**Keywords:** aneurysm, ischemic lesions, rupture, stroke, subarachnoid hemorrhage

## Abstract

Aneurysmal rupture concomitant with acute ischemic stroke (IS) unrelated to vasospasm has not been reported previously. We describe, for the first time, three cases of simultaneous aneurysmal subarachnoid hemorrhage and acute IS due to vessel occlusion located distally to the aneurysm. Two mechanisms are suggested for this concurrent occurrence of an aneurysm rupture and IS: (1) Occlusion of a blood vessel distal to the aneurysm from a clot originating from the aneurysm, thereby increasing intra-aneurysmal pressure and causing rupture; (2) rupture of a partially thrombosed aneurysm, resulting in the mobilization of a clot that causes vessel occlusion. Observation of such cases may enhance our understanding of the mechanisms involved in aneurysmal rupture.

## Introduction

Cerebral aneurysms are pathological dilatations of intracranial arteries that most commonly present with rupture, resulting in subarachnoid hemorrhage (SAH). SAH due to aneurysmal rupture is a medical emergency with high morbidity and mortality, necessitating rapid diagnosis and intervention. The worldwide incidence of aneurysmal SAH is estimated at approximately six cases per 100,000 person-years, though regional variations exist depending on genetic, environmental, and lifestyle factors [1].

While aneurysmal rupture leading to SAH is the most recognized clinical manifestation, cerebral aneurysms can also present with ischemic stroke (IS) or transient ischemic attacks. These events are believed to arise from embolic or thrombotic phenomena related to the aneurysm itself. Specifically, thrombus formation within the aneurysmal sac can lead to distal embolization or extend into the parent artery, causing vascular occlusion and downstream cerebral ischemia. Studies have reported that ischemic complications occur in approximately 3% of patients with unruptured intracranial aneurysms, most frequently involving the anterior circulation [2-6].

Even more unusual are cases where aneurysmal rupture is preceded by an ischemic event. This dual pathology presents a diagnostic and therapeutic challenge due to the overlapping neurological symptoms and the conflicting priorities in clinical management. Current literature on such cases remains sparse, consisting primarily of individual case reports and small case series [7-12].

We present a case series of patients who exhibited a simultaneous presentation of aneurysmal rupture and vascular occlusion, resulting in concurrent SAH and IS, without evidence of vasospasm. We explore the plausible pathophysiological mechanisms underlying this dual presentation and discuss its diagnostic and therapeutic implications.

## Case presentation

Case 1

**Figure 1:**
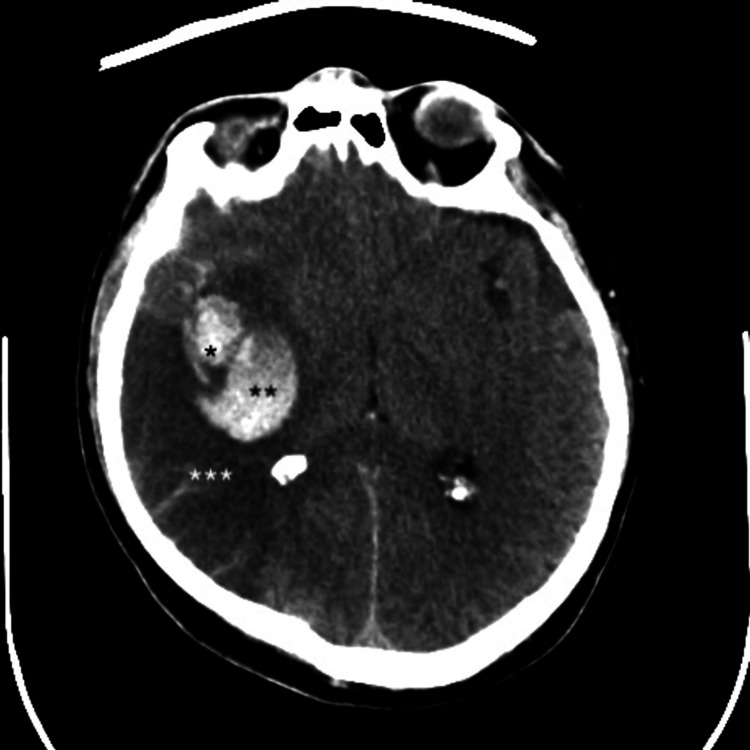
Axial brain CT scanner showing a subarachnoid hemorrhage associated with a right temporal hematoma, along with a right parieto-occipito-temporal junction ischemic stroke. *Partially thromboses M2M3 aneurism. **Right temporal hematoma. ***Ischemic lesion.

A 59-year-old man with no significant medical history other than hypertension presented with a sudden onset of severe headache on the day of admission. A brain CT scan revealed SAH associated with a right temporal hematoma (modified Fisher grade 4), along with a slight right hypodensity in the parieto-occipito-temporal junction (Figure 1). The patient was classified as Hunt and Hess (HH) grade 3. Neurological examination also revealed brachiofacial paresis (4/5) and left hemianopsia. Angio-CT and digital subtraction angiography demonstrated a small (7.5 mm) thrombosed aneurysm of the M2 segment of the middle cerebral artery (MCA) and occlusion of a parieto-occipital branch of the MCA. No vasospasm was identified. The aneurysm was surgically addressed, and post-operative CT confirmed the right MCA IS. The patient had a favorable post-operative outcome; however, a left superior quadrantanopia persisted.

Case 2

**Figure 2:**
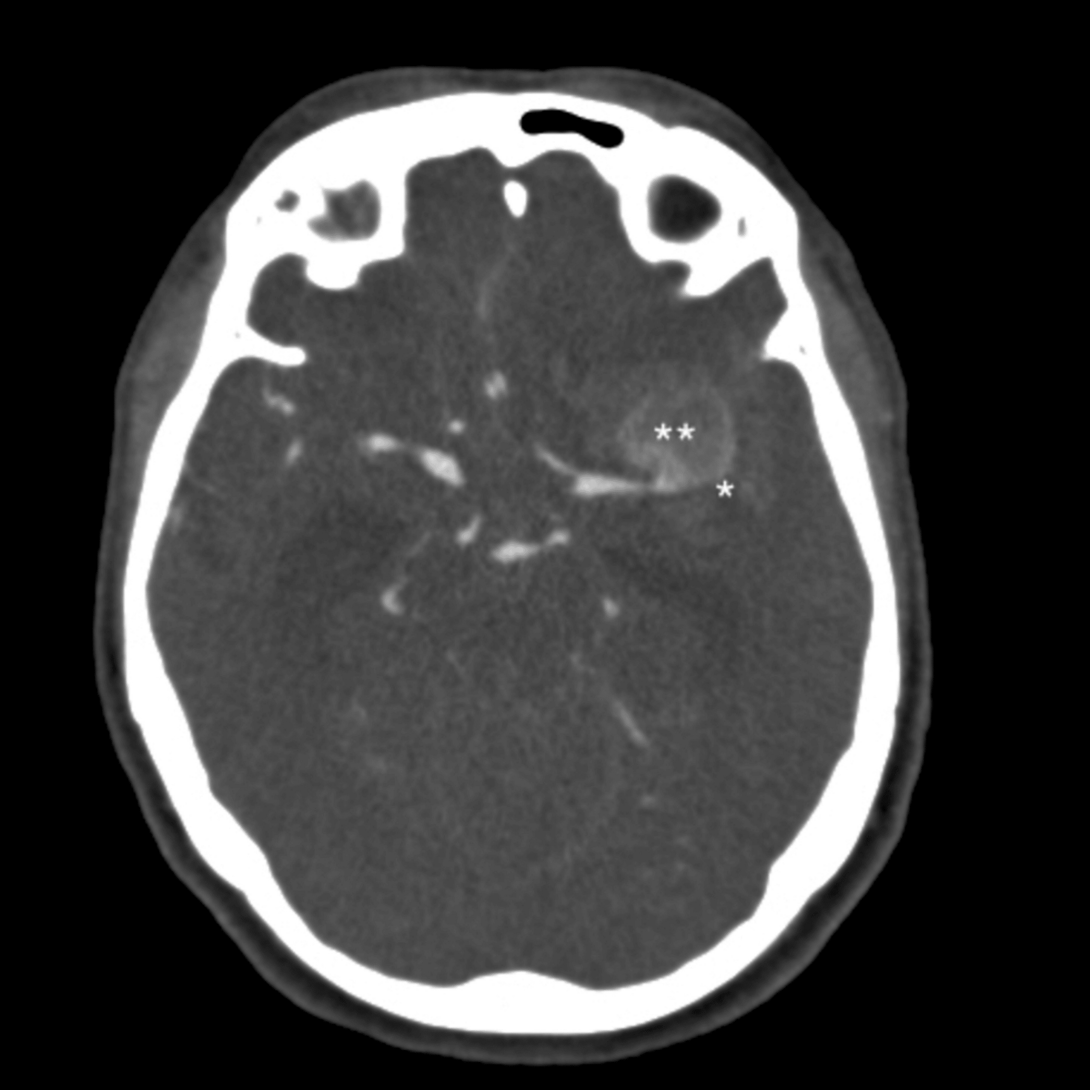
Axial brain CT scan with contrast reveals a subarachnoid hemorrhage secondary to the rupture of a left thrombosed middle cerebral artery bifurcation aneurysm, with occlusion of the M2 branches. *Occlusion of the M2 branches. **Left thrombosed MCA aneurism.

A 69-year-old woman lost consciousness at home and was admitted with HH grade 5 SAH and a left frontal hematoma (modified Fisher grade 4), resulting from the rupture of a giant MCA bifurcation aneurysm. CT angiography confirmed the presence of the giant MCA aneurysm and revealed occlusion of the distal MCA, which was responsible for the extensive IS (Figure 2). Cerebral angiography ruled out vasospasm and confirmed the distal MCA occlusion. Unfortunately, the patient died a few hours after admission as a result of intracranial hypertension secondary to the intraparenchymal hematoma and extensive MCA ischemia.

Case 3

**Figure 3:**
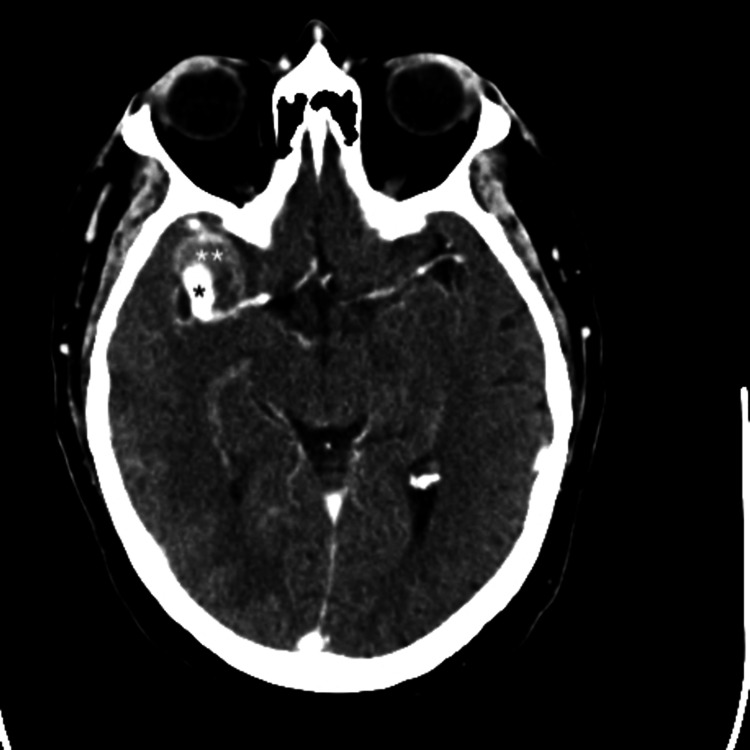
Axial CT scan with contrast shows a subarachnoid hemorrhage secondary to the rupture of a right partially thrombosed MCA bifurcation aneurysm. The ischemic stroke is not visible on this scan. *Circulating part of the MCA aneurysm. **Thrombosed part of the MCA aneurysm. MCA, middle cerebral artery

**Figure 4:**
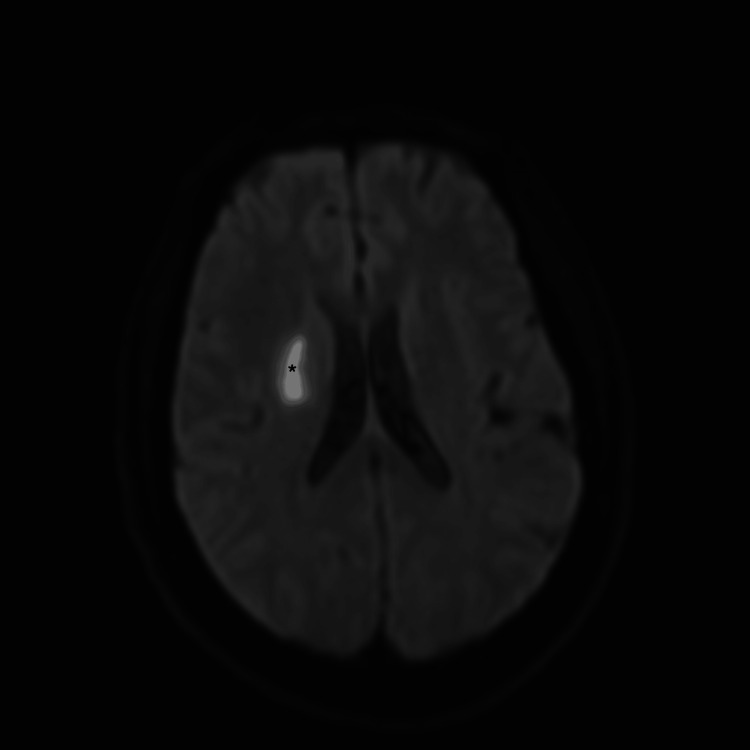
DWI MRI (B1000) revealing a deep MCA ischemic stroke. *Deep right ischemic stroke MCA, middle cerebral artery

A 56-year-old man with a medical history of hypertension, sleep apnea, and an IS related to a patent foramen ovale treated four years earlier presented with HH grade 2 SAH (modified Fisher grade 3) secondary to a giant partially thrombosed right MCA bifurcation aneurysm (Figure 3). A recent deep MCA IS was visible on the brain CT scan, and an MRI confirmed the presence of an IS (Figure 4). Because the aneurysm was deemed neither coilable nor clippable, we proceeded with a high-flow bypass followed by endovascular occlusion.

## Discussion

The neurological status following SAH is not always attributable solely to the hemorrhage itself or its direct consequences, such as hydrocephalus, elevated intracranial pressure, or intraparenchymal extension. In this series, we demonstrate that neurological deficits may also result from an IS unrelated to vasospasm. Importantly, vasospasm was ruled out in all patients through cerebral vessel angiography performed at the time of admission.

To our knowledge, this is the first report to describe the concomitant occurrence of SAH and IS following aneurysmal rupture in the absence of vasospasm. Although a limited number of case reports and small series have noted SAH as a complication arising early after IS distal to a thrombosed aneurysm, the simultaneous presentation of both SAH and IS at the time of aneurysmal rupture has not been previously documented [7-9]. An IS complicating a thrombosed aneurysm is therefore regarded by these authors as a warning sign for potential subsequent aneurysmal rupture, although the risk of this outcome may be quite low. The proposed mechanism involves subendothelial exposure, which can lead to thrombus formation and damage to the aneurysmal wall, ultimately resulting in rupture [7].

The common characteristic in our series is the presence of a thrombus in the affected aneurysm. While it is well known that unruptured aneurysms with a thrombus in their wall can lead to a stroke, this was not observed in our series [2-6].

Simultaneous ischemic infarcts and SAH are rarely described in arterial dissections without associated aneurysm [12]. No arterial dissection was observed in our cases. 

In our cases, the precise mechanism leading to concomitant rupture and stroke is not known. We propose the following two hypotheses.

One possibility is that the chemical activity within the thrombus weakens the aneurysm wall and lyses the intramural thrombus itself. This condition may lead to aneurysmal rupture and the release of a thrombus fragment, resulting in the occlusion of a distal branch. Thrombosis of aneurysms has been associated with factors such as calcification within the atherosclerotic wall, large- to giant-sized aneurysms, the ratio of chamber volume to orifice area, blood stagnation, increased blood viscosity, slow flow, and endothelial injury due to turbulent blood flow, which facilitates platelet deposition and aggregation [4,13,14].

The other hypothesis is that the dislodging of a clot from the thrombus causes occlusion of a distal branch with secondary increased distal resistance, which in turn produces increased intra-aneurysmal pressure leading to rupture. A sudden and brief increase in blood pressure seems to be a common trigger for aneurysmal rupture [14,15]. Since most of the aneurysms presenting with a stroke are unruptured, we assume that the wall of the aneurysm must already be weakened for the rupture to occur.

These hypotheses are not mutually exclusive, and these proposed mechanisms may occur simultaneously.

Regardless of which of these two mechanisms is at play, it is important to recognize the occurrence of a concomitant rupture and stroke since the treatment of these aneurysms presents increased risk, whether by coiling or clipping [8].

Recognizing the associated IS before treatment is essential since ignorance could lead to considering the IS as a consequence of the treatment.

## Conclusions

Ischemia occurring alongside aneurysmal rupture and SAH is not always attributable to vasospasm. This article presents alternative mechanisms for acute ischemia that occur simultaneously with SAH. Although rare, this synchronous occurrence of aneurysmal rupture and IS represents a critical clinical scenario requiring prompt recognition and thoughtful management. This dual presentation can complicate diagnosis due to overlapping neurological signs and necessitates a careful therapeutic balance between mitigating ongoing hemorrhage and addressing cerebral ischemia. Understanding the underlying mechanisms, such as thromboembolic events and compromise of the vascular wall, is essential for guiding timely and effective interventions. The complexity of these cases highlights the need for advanced imaging, multidisciplinary collaboration, and individualized treatment strategies. Raising awareness of this presentation among clinicians can facilitate earlier diagnosis and improve patient outcomes, underscoring the importance of continued focus on this area in clinical practice.
